# Baseline prevalence and intensity of schistosomiasis at sentinel sites in Madagascar: Informing a national control strategy

**DOI:** 10.1186/s13071-016-1337-4

**Published:** 2016-01-27

**Authors:** Clara Fabienne Rasoamanamihaja, Alain Marcel Rahetilahy, Bruno Ranjatoarivony, Neerav Dhanani, Luciano Andriamaro, Samuel Hermas Andrianarisoa, Peter Mark Jourdan

**Affiliations:** Ministry of Public Health, Antananarivo, Madagascar; Ministry of National Education, Antananarivo, Madagascar; Schistosomiasis Control Initiative (SCI), Imperial College London, London, UK; Réseau International Schistosomose Environnement Aménagement et Lutte (RISEAL) Madagascar, Antananarivo, Madagascar; World Health Organization (WHO) Madagascar, Antananarivo, Madagascar

**Keywords:** Madagascar, Preventive chemotherapy, Schistosomiasis, *Schistosoma haematobium*, *Schistosoma mansoni*, Soil-transmitted helminths, Non-attending school-age children, Water, Sanitation and hygiene

## Abstract

**Background:**

Schistosomiasis affects more than 800 million people, mostly in sub-Saharan Africa. A baseline sentinel site study was conducted in the Western half of Madagascar to determine the prevalence and intensity of schistosomiasis and soil-transmitted helminth (STH) infections prior to mass drug administration, and to explore the associations between infection and school attendance, and access to water, sanitation and hygiene (WASH) facilities.

**Methods:**

A three-stage, cluster-randomised cross-sectional study was conducted in 29 sentinel sites in October 2015. Twenty school attending and 4 non-attending children in each of the age groups from 7 to 10 years old were randomly selected at each site for detection of *Schistosoma haematobium* eggs in a single urine slide by filtration, and of *S. mansoni*, *Ascaris lumbricoides*, *Trichuris trichiura* and hookworm eggs in duplicate Kato-Katz slides from a single stool sample. School attendance was registered individually, and school-level access to WASH facilities was scored through pre-defined observed and reported factors. Logistic regression analysis was performed, adjusting for gender, age and study site. School-level WASH status was analysed using Spearman’s rank correlation coefficient.

**Results:**

A total of 1,958 children were included. The prevalence of *S. haematobium* infection and heavy-intensity infection was 30.5 % and 15.1 %, respectively. The prevalence of *S. mansoni* infection and heavy-intensity infection was 5.0 % and 0.9 %, respectively. The prevalence of any STH infection was 4.7 %. There was no significant difference in prevalence of infection or heavy-intensity infection of either schistosome species between attending and non-attending children, apart from heavy-intensity *S. mansoni* infection that was significantly more common in children who did not attend school regularly (aOR = 7.5 (95 % CI = 1.1-49.5); *p* = 0.037). Only a minority of schools had adequate access to WASH facilities, and in this study, we found no significant association between school-level WASH status and schistosomiasis.

**Conclusions:**

This study found an alarmingly high prevalence and intensity of schistosomiasis, and the results warrant urgent scale-up of the national NTD control programme that will need to include both non-attending and attending school-age children in order to reach WHO roadmap targets for the control of schistosomiasis by 2020.

**Electronic supplementary material:**

The online version of this article (doi:10.1186/s13071-016-1337-4) contains supplementary material, which is available to authorized users.

## Background

Schistosomiasis is a waterborne blood fluke infection that affects more than 800 million people globally, and more than 90 % live in sub-Saharan African countries with poor access to clean water and sanitary facilities [1]. Despite schistosomiasis being one of the leading causes of impaired health and socio-economic development in the world, effective, safe and freely donated treatment exists and may contribute to control disease through large-scale preventive chemotherapy (PC) recommended by the World Health Organization (WHO) [2].

More than 6.8 million individuals in Madagascar, nearly half of them school-age children (SAC), are in need of PC for schistosomiasis; however, in 2014 the national coverage of praziquantel was only 27 % [3]. In 2014, the Ministry of Public Health of Madagascar launched the national Neglected Tropical Disease (NTD) Master Plan for 2014-2018 [4]. In collaboration with the Ministry of National Education, WHO, technical and financial partners, the Ministry of Public Health currently targets five endemic NTDs through PC: schistosomiasis, soil-transmitted helminth (STH) infections and lymphatic filariasis (LF). Close to a third of the districts in the Western half of Madagascar targeted for treatment through the support of the Schistosomiasis Control Initiative (SCI) have never previously received PC, and nearly two thirds have not been offered treatment for 5 years. The SCI-supported intervention area is limited by available resources for drug distribution, and complements on-going NTD control in Southern Madagascar funded by the World Bank.

Monitoring and evaluation of prevalence and intensity of infection are recommended in order to document the impact of treatment, and to improve control programmes when necessary [5]. In line with WHO guidelines, a baseline study of sentinel sites was conducted in order to determine the prevalence and intensity of schistosomiasis prior to mass drug administration. Moreover, this study aims to explore the association between infection and school attendance, and between infection and access to water, sanitation and hygiene (WASH) facilities at schools. This paper reports on the prevalence and intensity of schistosomiasis in areas left untreated for at least half a decade, in relation to gender, age, school attendance and access to adequate WASH facilities.

## Methods

### Study design and setting

A three-stage, cluster-randomised cross-sectional study was conducted in the Western half of Madagascar in October 2015 to determine the prevalence and intensity of schistosomiasis in sentinel sites at baseline prior to mass PC with praziquantel. The study sites are located in 3 main ecological zones; humid, sub-humid and sub-arid zones as defined by humidity and temperature [6]. The majority of the population are farmers who rely on income mainly from rice cultivation and animal husbandry [7]. Nearly half of Madagascar’s population is younger than 15 years old, and the study area, is home to about 1.5 million SAC 5 to 14 years old [8]. The primary school enrolment ratio in Madagascar is similar to the average for sub-Saharan Africa [9]; however, in more than half the study area, a report suggests that less than 55 % of SAC attend school regularly [7].

### Study participants

In order to draw a representative sample from the population targeted for treatment, all children at the study sites aged 7 to 10 years old were invited for selection, regardless of whether they were officially enrolled in school or attended school regularly or not. Ten girls and 10 boys who attended school regularly, and 2 girls and 2 boys who did not attend school regularly were targeted for random selection in each of the 4 age groups in order to account for any differences in prevalence and intensity of infection between age groups and between sites. In sites where the targeted number of attending and non-attending children were not present, all eligible children aged 7 to 10 years were included. The head of village and head teacher of each site were contacted the day prior to the study in order to ensure that as many as possible of the children were present; however, the targeted numbers of attending and non-attending children were not present at all sites. The age range was chosen in order to ensure a sufficient sample size of appropriate age groups according to WHO guidelines [5]. Mass drug administration of praziquantel had not taken place in any of the study sites at least 5 years prior to the study.

### Parasitological data

Stool and urine samples were collected in sterile plastic pots. Each included child was allocated a unique identification number that was attached to the stool and urine pots and noted on the registration and laboratory data collection sheets.

Two slides per individual stool sample were prepared by filling a Kato-Katz template on 2 separate slides with stool (Kato-Katz kit, Vestergaard-Frandsen, Lausanne, Switzerland), levelling and covering each sample with a cellophane slip pre-stained with methylene blue [10]. The stool slides were read within 60 minutes for detection of hookworm eggs, and, after adequate clarification for detection of *Schistosoma mansoni*, *Ascaris lumbricoides* and *Trichuris trichiura* eggs.

Urine was sampled between 10 am and 2 pm for optimal detection of urinary *Schistosoma* eggs. One slide per individual was prepared by shaking the pot of urine, drawing 10 mL, or if less than 10 mL the whole quantity of urine, from the sample pot and passing the urine through a filter placed in a pre-assembled filter holder (Sterlitech Corporation, Kent, USA). A drop of Lugol’s solution was added to facilitate identification and counting of eggs.

The slides were examined by conventional light microscopy (Novex, Euromex, the Netherlands) by 2 officially certified laboratory technicians per site. The technicians were selected from a group of laboratory technicians evaluated and selected by 2 medical doctors with specialist training in parasitological laboratory diagnosis. Any uncertainties of slide interpretation were discussed and resolved on site by consensus between the 2 laboratory technicians. The crude parasitological results were registered alongside the respective unique identification numbers on the laboratory data collection sheets. The number of eggs per gram (epg) of stool was calculated for each of the examined species by multiplying the crude egg number per slide by 24 in line with producer instructions. Heavy-intensity infection was defined as >399 *S. mansoni* epg, >49,999 *A. lumbricoides* epg, >3,999 hookworm epg, and >9,999 *T. trichiura* epg, respectively [5]. The number of *S. haematobium* eggs per 10 mL of urine was calculated by multiplying the crude egg numbers per slide with the number of mL of the respective urine sample and dividing by 10. Heavy-intensity infection was defined as >50 *S. haematobium* eggs per 10 mL [5].

### School attendance

The status of school attendance as defined by the head teacher at each school was registered alongside each child’s parasitological data. “Regular” school attendance was defined as attendance during most teaching days, whereas “not regular” school attendance was defined as non-attendance during most teaching days.

### Water, sanitation and hygiene (WASH) status at schools

At each school, the WASH status was registered through observations made by the study team and conditions reported by the children and teachers. The parameters were selected from a set of priority factors defined by NTD and WASH organisations in a recent online consultation [11], and included access to an improved water source; functional, clean and adequate number of latrines; hand washing stations with soap; and hygiene teaching at the school. A WASH score was calculated by adding the results at each school; a minimum score of 0 meant no factors were present and a maximum score of 12 indicated full access to adequate WASH facilities.

### Power calculation, sampling strategy and statistical analysis

Sample size calculations suggest that, with 95 % significance and 80 % power, and assuming an intra-class correlation of 0.05, at least 30 children each in 29 sites will allow a detection of change in prevalence of schistosomiasis of at least 10 % from an estimated initial prevalence of 63 % (mean prevalence from available mapping data in the study area) in follow-up studies at the selected sentinel sites. The study design effect was estimated at 2.5 and was accounted for in the sample size estimation. This sample size equates to one site per 50,000 SAC in the treatment area, which is substantially higher than the WHO recommendation of one site per every 200,000 to 300,000 targeted children. The proportion of non-attending SAC was estimated to provide a logistically manageable sample (dividable by a factor of 8 to ensure equal numbers of boys and girls included in each of the 4 age groups) and that would reflect the national school attendance ratio as closely as possible [12].

**Fig. 1:**
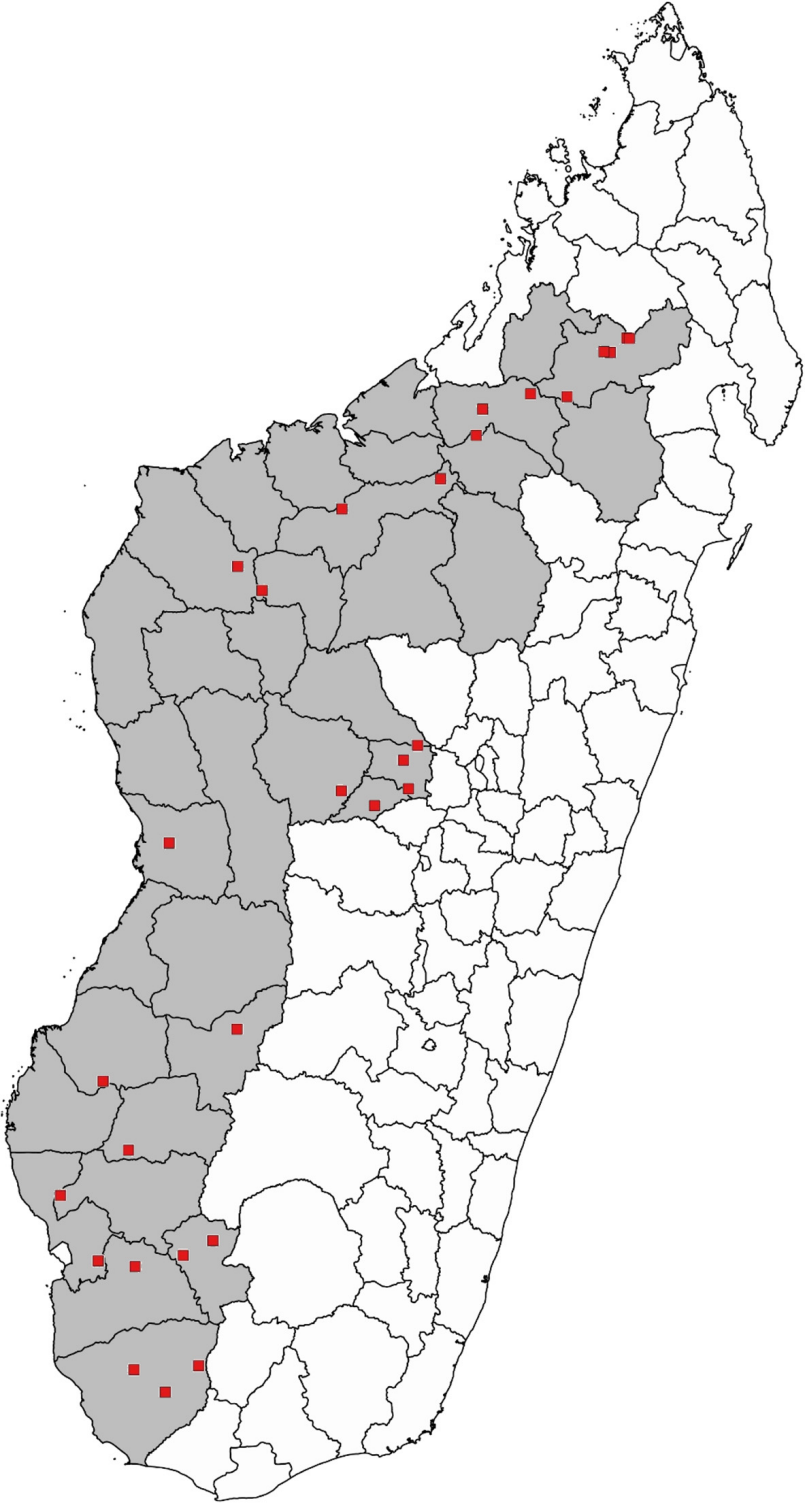
The study sites in the targeted treatment area in the Western half of Madagascar. A total of 29 sites (red) were selected through a three-stage, cluster-randomisation from public primary schools in an area in the Western half of Madagascar (grey) targeted for preventive chemotherapy through the support of the Schistosomiasis Control Initiative (SCI)

A three-stage cluster sampling was done of districts (1), villages (2) and households (3). Up-to-date prevalence data for schistosomiasis was not available for 19 study districts at the time of the planning of this study, and for the purposes of site selection these were treated as ‘unmapped’. Historical mapping data indicate that the prevalence of schistosomiasis in these districts is at least moderate according to WHO risk categories [5]. A total of 29 sites (public primary schools) were randomly selected from a list of all public primary schools; 10 study sites were selected from mapped districts and a further 19 from unmapped districts (Fig. 1). Mapped districts were stratified by prevalence categories (high/low, moderate/low, low/high, low/moderate and high/moderate for *S. mansoni*/*S. haematobium*). Unmapped districts were not stratified, and sites were randomly selected from a list of all public primary schools in these districts. Sites treated in a pilot project in 2014 were excluded from the list of schools to be selected as study sites.

Data were entered into Microsoft Excel (Microsoft Corporation, Redmond, USA) and analysed using the Statistical Package for the Social Sciences (SPSS) version 22 (IBM Corporation, New York, USA). Schistosome and STH egg counts were calculated using the arithmetic mean. Bivariate analysis was performed by including one covariate at a time in a logistic regression model with prevalence of infection and prevalence of heavy-intensity infection as binary dependent variables. In order to study the effect of several variables simultaneously, multivariate logistic regression analysis was performed on dependent variables of prevalence of infection and prevalence of heavy-intensity infection. Gender, age, school attendance and study sites were included as fixed categorical variables, and the WASH score was included as a linear variable.

Possible correlation between school-level parasitological data and WASH status were analysed using Spearman’s rank correlation coefficient as the parasitological parameters were not normally distributed. These analyses were done on the school-level mean for regular school attenders only as the WASH parameters were specifically collected for attending SAC.

Separate analyses were performed on the subset of sites with moderate to high prevalence of *S. haematobium* and *S. mansoni*, respectively, in order to evaluate the correlates in areas where the force of transmission was expected to be high. A 5 % significance level was used throughout.

### Ethics, consent and permissions

This study was granted ethical approval by the National Ethics Committee of Madagascar (N°121 MSANP/CE). The local health, educational and administrative authorities, and any parents present were comprehensively informed about the study, and any questions were answered and discussed prior to the study taking place. All were informed in the local language Malagasy, and any child could withdraw from the study at any point in time, without any consequences for the child or the person making such a decision. The head teacher was invited to provide free and informed, written consent on behalf of the children to allow the collection and analysis of stool and urine samples.

As all children included in the study were to be targeted for treatment of schistosomiasis and STHs in December 2015, and in order to enable unbiased monitoring of the effect of the National NTD control programme, the children were not treated for infections diagnosed during the study. A list of the children’s names was registered and kept confidentially by the Ministry of Public Health, in case some children were not treated due to any unforeseen circumstances. All other data were treated de-anonymised, and the list of the included children’s names will be discarded once treatment has been confirmed through official treatment reports.

## Results

A total of 1,958 children from 29 sites across 18 districts were included in the study, covering an area of more than 135,000 km^2^. The estimated sample size of at least 30 children was included at each site. Overall, 1,946 children provided a single day urine sample, and 1,934 children provided a single day stool sample for duplicate slide preparation. All children had lived at least 5 years in the respective study sites, and the majority (99.5 %, *n* = 1,948) had lived there for the duration of their lives. Table 1 summarises the study population characteristics, school attendance, schistosomiasis and STH infections. The prevalence of STH infections was low (4.7 %) and will be discussed elsewhere.

**Table 1:** Study population characteristics, schistosomiasis and STHs in 29 sentinel sites in the Western half of Madagascar

Study population characteristic and infection	*n* (%), mean (95 % CI)
Gender	
Male	915 (46.7)
Female	1,043 (53.3)
Age	
7 years	532 (27.2)
8 years	495 (25.3)
9 years	474 (24.2)
10 years	457 (23.3)
School attendance	
Regular	1,606 (82.0)
Not regular	352 (18.0)
Schistosomiasis	
Any schistosomiasis	684 (35.3)
*S. haematobium* infection	594 (30.5)
*S. haematobium* heavy-intensity infection	294 (15.1)
Mean *S. haematobium* egg count	62.2 (95 % CI 49.0–75.3)
*S. mansoni* infection	97 (5.0)
*S. mansoni* heavy-intensity infection	17 (0.9)
Mean *S. mansoni* egg count	14.2 (95 % CI 7.7–20.6)
Mixed schistosome infection	7 (0.5)
Soil-transmitted helminth (STH) infections	
Any STH infection	90 (4.7)
*A. lumbricoides* infection	86 (4.4)
Mean *A. lumbricoides* egg count	116.8 (95 % CI 58.9–174.7)
*T. trichiura* infection	43 (2.2)
Mean *T. trichiura* egg count	1.0 (95 % CI 0.3–1.6)
Hookworm infection	62 (3.2)
Mean hookworm egg count	2.3 (95 % CI 0.9–3.7)

School attendance = as reported by the head teacher of the respective school; *S. haematobium* infection = *Schistosoma haematobium* eggs identified by urine filtration; prevalence of heavy-intensity *S. haematobium infection* = >50 *S. haematobium* eggs per 10 mL of urine; *S. mansoni* infection = *S. mansoni* eggs identified by duplicate Kato-Katz slides; prevalence of heavy-intensity *S. mansoni infection* = >399 *S. mansoni* eggs per gram of stool (epg); mean (95 % CI) = arithmetic mean (95 % confidence interval); *A. lumbricoides* infection = *A. lumbricoides* eggs identified by duplicate Kato-Katz slides; prevalence of heavy-intensity *A. lumbricoides infection* = >49,999 *A. lumbricoides* epg of stool; *Trichuris trichiura* infection = *T. trichiura* eggs identified by duplicate Kato-Katz slides; prevalence of heavy-intensity *T. trichiura infection* = >9,999 *T. trichiura* epg of stool; Hookworm infection = hookworm eggs identified by duplicate Kato-Katz slides; prevalence of heavy-intensity hookworm *infection* = >3,999 hookworm epg of stool

### Schistosomiasis prevalence and intensity of infection

The overall prevalence of *S. haematobium* and *S. mansoni* infection in the study area was 30.5 % and 5.0 %, respectively, and the prevalence of heavy-intensity infection was 15.1 % and 0.9 %, respectively. Only 7 (0.5 %) cases presented with mixed schistosome infections with *S. haematobium* and *S. mansoni*. Tables 2 and 3 show the prevalence of infection and heavy-intensity infection in relation to gender, age, school attendance and study sites. In bivariate analysis, neither the prevalence of *S. haematobium* nor the prevalence of *S. mansoni* infection varied significantly by gender or age. The percentage of heavy-intensity *S. mansoni* infection was significantly lower in 10 year olds compared to 7 year old children; otherwise there was no association between heavy-intensity *S. haematobium* or *S. mansoni* infection and gender or age.

**Table 2:** School attendance and schistosomiasis *haematobia* in 29 sentinel sites in the Western half of Madagascar

	Prevalence of *S. haematobium* infection						Prevalence of heavy-intensity *S. haematobium* infection			
	*n*	mean prevalence	OR	*p*	aOR	*p*	OR	*p*	aOR	*p*
			(95 % CI)		(95 % CI)		(95 % CI)		(95 % CI)	
Gender										
Male	269	29.6								
Female	325	29.6	1.1 (0.9–1.3)	0.39	1.1 (0.8–1.4)	0.45	0.9 (0.7–1.2)	0.57	0.9 (0.7–1.2)	0.52
Age										
7 years	142	26.9								
8 years	156	31.8	1.3 (1.0–1.7)	0.087	1.2 (0.8–1.7)	0.40	1.0 (0.7–1.4)	0.83	0.7 (0.5–1.1)	0.18
9 years	153	32.5	1.3 (1.0–1.7)	0.053	1.5 (1.0–2.1)	0.039	1.0 (0.7–1.4)	0.83	0.8 (0.5–1.3)	0.44
10 years	143	31.4	1.2 (0.9–1.6)	0.12	1.3 (0.9–1.9)	0.19	1.0 (0.7–1.4)	1.0	0.9 (0.6–1.4)	0.62
Study site				<0.001		<0.001		<0.001		<0.001
School attendance										
Regular	501	31.4								
Not regular	93	26.6	0.8 (0.6-1.0)	0.083	0.7 (0.5-1.0)	0.075	1.0 (0.7-1.3)	0.78	1.2 (0.8-1.8)	0.42

*S. haematobium* infection = *Schistosoma haematobium* eggs identified by urine filtration; prevalence of heavy-intensity *S. haematobium infection* = >50 *S. haematobium* eggs per 10 mL of urine; *OR* odds ratio (bivariate analysis), *aOR* adjusted OR (multivariate analysis), *CI* confidence interval; school attendance = as reported by the head teacher of the respective school; study site = selected public primary schools across an area of 36 districts in Madagascar targeted for control of schistosomiasis with the support of SCI

**Table 3:** School attendance and schistosomiasis *mansoni* across 29 sentinel sites in the Western half of Madagascar

	Prevalence of *S. mansoni* infection						Prevalence of heavy-intensity *S. mansoni* infection^a^			
	*n*	mean prevalence	OR	*p*	aOR	*p*	OR	*p*	aOR	*p*
			(95 % CI)		(95 % CI)		(95 % CI)		(95 % CI)	
Gender										
Male	50	5.5								
Female	47	4.6	0.8 (0.5–1.2)	0.33	0.8 (0.4–1.3)	0.33	1.0 (0.4–2.6)	0.98	1.4 (0.4–5.2)	0.60
Age										
7 years	28	5.4								
8 years	19	3.9	0.7 (0.4–1.3)	0.26	0.7 (0.3–1.5)	0.34	0.2 (0.1–1.1)	0.063	0.2 (0.0-1.4)	0.12
9 years	25	5.3	1.0 (0.6–1.7)	0.96	1.1 (0.5–2.3)	0.78	0.6 (0.2–1.8)	0.38	0.6 (0.1-2.5)	0.48
10 years	25	5.5	1.0 (0.6–1.8)	0.93	1.5 (0.8–3.0)	0.25	0.1 (0.0–1.0)	0.049	0.2 (0.0-2.0)	0.17
Study site				<0.001		<0.001		^b^		^b^
School attendance										
Regular	79	5.0								
Not regular	18	5.2	1.1 (0.6-1.8)	0.85	1.7 (0.9-3.4)	0.12	1.9 (0.7-5.5)	0.22	7.5 (1.1-49.5)	0.037

*S. mansoni* infection = *S. mansoni* eggs identified by duplicate Kato-Katz slides; prevalence of heavy-intensity *S. mansoni infection* = >399 *S. mansoni* eggs per gram of stool; *OR* odds ratio (bivariate analysis), *aOR* adjusted OR (multivariate analysis), *CI* confidence interval; school attendance = as reported by the head teacher of the respective school; ^a^Analysis performed on 17 cases only; ^b^Analysis of prevalence of heavy-intensity *Sn mansoni* infection across study sites could not be performed due to low sample size; study site = selected public primary schools across an area of 36 districts in Madagascar targeted for control of schistosomiasis with the support of SCI

### Schistosomiasis and school attendance

**Fig. 2:**
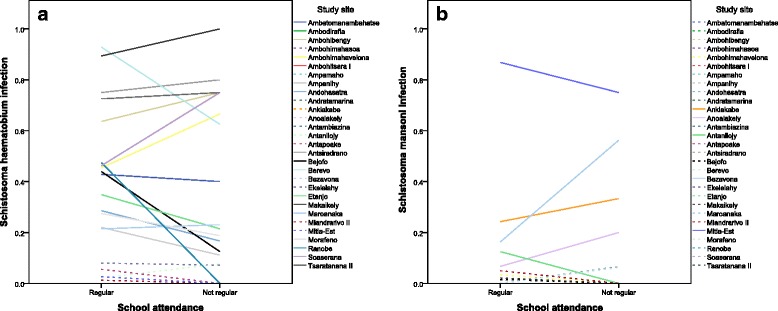
School attendance and prevalence of *S. haematobium* (**a**) and *S. mansoni* (**b**) infection. There was no significant association between school attendance and prevalence of *S. haematobium* or *S. mansoni* infection across 29 sentinel sites in the Western half of Madagascar. Dotted lines indicate sites with low prevalence of infection (<10 % per WHO categories) of the respective species. Note that sites with 0 % prevalence overlap with the x-axis. *Schistosoma* *haematobium* infection = ≥1 *Schistosoma haematobium* egg(s) identified by urine filtration; School attendance = as reported by the head teacher at the school; *Schistosoma* *mansoni* infection = ≥1 *S. mansoni* egg(s) identified by duplicate Kato-Katz slides

Figure 2 shows the prevalence of *S. haematobium* (A) and *S. mansoni* (B) according to school attendance at each site. Further details of the site-specific prevalence of *S. haematobium *and *S. mansoni *are give in Additional file 1: Tables S1 and S2, respectively. In bivariate analysis, there was no significant difference in prevalence of *S. haematobium* or *S. mansoni* infection between children who attended and who did not attend school regularly (odds ratio (OR) = 0.8 (95 % confidence interval (CI) = 0.6-1.0); *p* = 0.083 and OR = 1.1 (95 % CI = 0.6-1.8); *p* = 0.85, respectively). As shown in Tables 2 and 3, when adjusting for gender, age and study site in multivariate analysis, the association between school attendance and infection with either schistosome species remained insignificant. The prevalence of infections with both species varied significantly across study sites. In the 2 sites with heavy-intensity *S. mansoni* infection, children who did not attend school regularly had a significantly higher prevalence than attending children (OR = 7.5 (95 % CI = 1.1-49.5); *p* = 0.037). No other significant association was found between school attendance and prevalence of heavy-intensity infection of either species.

In multivariate analysis of sites with moderate to high prevalence of *S. haematobium* infection only, there was no significant difference in prevalence of infection between children who attended and who did not attend school regularly, when adjusting for gender, age and study sites (adjusted (a)OR = 0.7 (95 % CI = 0.5-1.0); *p* = 0.063) or prevalence of heavy-intensity infection (aOR = 1.2 (95 % CI = 0.8-1.8); *p* = 0.39). Multivariate analysis of sites with moderate to high prevalence of *S. mansoni* infection found a significantly higher prevalence of both infection and heavy-intensity infection in children who did not attend school regularly (aOR = 2.7 (95 % CI = 1.2–6.1); *p* = 0.019 and aOR = 7.5 (95 % CI = 1.1–49.5); *p* = 0.037, respectively).

### Schistosomiasis and access to WASH facilities at schools

**Fig. 3:**
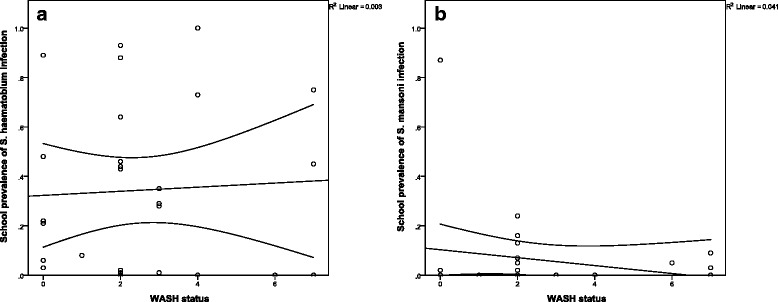
*S. haematobium* (**a**) and *S. mansoni* (**b**) infection according to WASH status. There was no significant association between school prevalence of *S. haematobium* (**a**) or *S. mansoni* (**b**) and overall WASH status at schools. Analyses were performed on the school-level mean for attending children only as the WASH parameters were specifically collected for attending school-age children. *S. haematobium* infection = ≥1 *Schistosoma haematobium* egg(s) identified by urine filtration; *S. mansoni* infection = ≥1 *S. mansoni* egg(s) identified by duplicate Kato-Katz slides; Any STH infection = ≥1 *Ascaris lumbricoides*, *Trichuris trichiura* and/or hookworm egg(s) identified by duplicate Kato-Katz slides; WASH status = total score of observed and reported access to an improved water source; functional, clean and adequate number of latrines; hand washing stations with soap; and hygiene teaching at the school - a minimum score of 0 indicated that no factors were present and a maximum score of 12 that all factors were present. The central line indicates best fit of the correlation between the 2 variables, and the 2 curved lines indicate the 95 % confidence interval of the best fit line

Seventy-five per cent of schools had a WASH score <3, and the maximum score was 7, indicating an especially limited access to WASH facilities at schools in the study area. Figure 3 shows the mean school-level prevalence of *S. haematobium* infection (A) and *S. mansoni* infection (B) according to the WASH status. There was no significant association between WASH status at schools and prevalence of *S. haematobium* or *S. mansoni* infection (*p* = 0.87 and *p* = 0.92, respectively) or prevalence of heavy-intensity *S. haematobium* or S. mansoni infection (*p* = 0.70 and *p* = 0.23, respectively). These associations remained insignificant in sites with moderate to high prevalence of *S. haematobium* and *S. mansoni* (*p* = 0.82 and *p* = 0.48, and *p* = 0.23 and *p* = 0.18, respectively).

## Discussion

Neglected tropical diseases, including schistosomiasis, are a major public health problem in Madagascar. Despite WHO providing praziquantel free of charge for every school-age child in need of treatment, less than half of Madagascar’s at-risk school-age population is currently being treated [3]. This study identified a high prevalence of especially urogenital schistosomiasis in the Western half of Madagascar, and there is a critical and urgent need to scale up and sustain national control measures for schistosomiasis. In an era when the global health community is preparing to target the elimination of schistosomiasis as a public health problem in selected countries, the findings of this study indicate an alarmingly high prevalence of schistosomiasis in areas where the population at risk has been neglected for several years [13].

Studies have reported baseline sentinel site results from limited geographical areas elsewhere [14–16], and from a selection of sentinel sites that was purposively rather than randomly selected [16, 17]. Large-scale sentinel site studies for the monitoring and evaluation of the national NTD control programme in Burkina Faso found an overall prevalence of *S. haematobium* infection of less than half of that found in this study [18], and studies in Kenya and Tanzania reported a similarly low to moderate overall prevalence of *S. haemtobium* and *S. mansoni* infection, respectively [19, 20].

Large-scale surveys determining the prevalence of schistosomiasis in Madagascar have been scarce, and data has been derived from historical records or individual studies [21, 22]. Several studies have highlighted the occurrence of genital *S. haematobium* infection that may represent an unrecognised burden of disease in women and men [23, 24]. The recent WHO-led mapping surveys will address the lack of updated, nationwide prevalence data of schistosomiasis, and ensure appropriate resource allocation for NTD control in Madagascar.

Few mixed schistosome infections were identified in this study, possibly due to the focal distribution of intermediate snail hosts and limited human migration between transmission sites [25]. In line with current WHO recommendations, data on pre-SAC was not collected as part of this study [5]. Praziquantel is not currently approved for use in pre-SAC, and models suggest that treating pre-SAC might not substantially contribute to the elimination of schistosomiasis as a public health problem [26]. Overall, STHs were not common in this study area; however, disease prevalence was highly focal and needs to be addressed through appropriate NTD control measures.

In line with our findings, studies have found an equally high or higher prevalence of schistosomiasis in SAC who do not attend school regularly [27–30]. Some reports suggest that school attendance decreases with increasing intensity of infection, making intensified efforts to reach non-attending SAC all the more important [31]. Schools may be a convenient platform for large-scale preventive chemotherapy for schistosomiasis, and is currently the main strategy recommended by WHO [5, 32, 33]. However, as the control of schistosomiasis is lagging behind other NTD control programmes [34], studies suggest that current school-based treatment programmes may not be sufficient for the control of disease and reduction of morbidity [26, 35–39]. The reportedly low primary school attendance in parts of the study area may prevent non-attending SAC from being treated through school-based PC, and alternative strategies are needed to effectively reach SAC who do not attend school regularly [40].

This study has some limitations. Firstly, complete mapping data was not available prior to the study, and the sample size estimation could therefore not take into account the prevalence of the respective schistosome species. The sample size was calculated for the purpose of detecting a change in prevalence of schistosomiasis, and the study might not have had sufficient power to draw conclusions with regard to other variables. Further, at some sites, the remote location limited the sample size and the possibility of conveniently increasing sampling by recruitment of children from a neighbouring village. This also restricted the number of non-attending SAC in some sites; however, post-hoc sample size calculation showed that the included sample of non-attending and attending children did not affect the detectable range of difference in schistosomiasis between the 2 groups. Secondly, although this study followed WHO guidelines for diagnosis of *S. mansoni* and STHs, studies have shown that Kato-Katz technique may have a low sensitivity for *S. mansoni* and STHs, although duplicate slides of a single stool sample may be sufficient at baseline in moderate and high prevalence areas [41, 42]. Finally, this study was cross-sectional and conclusions cannot be drawn with regard to causality of associations.

This study demonstrated the feasibility of integrating the collection of WASH parameters as part of an NTD monitoring and evaluation study. Although the limited access to WASH facilities in the study area may have prevented detection of an association between access to WASH and schistosomiasis, it is possible that the indicators need to be evaluated to define their validity in accurately determining their impact on schistosomiasis. The particularly limited access to WASH facilities in the study area indicates a critical need to provide safe drinking water, adequate sanitation and hygiene facilities in parallel with the scale-up of other NTD control measures [43]. WHO has recently published a joint NTD and WASH strategy that urges stakeholders to improve advocacy, monitoring, evidence-generation and delivery of effective WASH interventions [44].

Recent analyses of the effect of WASH on schistosomiasis indicate that faecal helminth infections are more common in areas with limited access to WASH facilities [45]. In addition to the health benefits, one study demonstrated that providing adequate WASH facilities may benefit SAC in terms of enrolment and gender equity [46]. Few randomised-controlled trials have evaluated the effect of WASH on schistosomiasis, and the efforts to date have been limited by methodological challenges, including delivery of effective interventions, study design and definition of valid outcome measures [47, 48].

## Conclusions

This study identified an alarmingly high prevalence and intensity of schistosomiasis, especially of urogenital *S. haematobium* infection, in a population that has been left untreated for at least half a decade. The findings suggest that the national NTD control programme needs to target both non-attending and attending SAC as a substantial portion of infection is harboured by children in both groups. This study demonstrated the feasibility, but also potentially the need to validate, the integrated collection of WASH data in national NTD control surveys, and the need to address the lack of access to adequate WASH facilities in the study area. In order to reach WHO roadmap targets for control of schistosomiasis as a public health problem by 2020, intensified scale-up and sustained, integrated control of schistosomiasis and other NTDs in Madagascar is urgently needed.

## Additional file

Additional file 1: Table S1. Prevalence of *S. haematobium* infection according to school attendance in the respective study sites. **Table S2.** Prevalence of *S. mansoni* infection according to school attendance in the respective study sites. (DOCX 15 kb)
