# Supplementary material for: Baseline prevalence and intensity of schistosomiasis at sentinel sites in Madagascar: Informing a national control strategy
Source: Parasit Vectors. 2016 Jan 27;9:50. doi: 10.1186/s13071-016-1337-4 (PMC4730633; doi:10.1186/s13071-016-1337-4)
Supplement: Additional file 1: Table S1. — Prevalence of S. haematobium infection according to school attendance in the respective study sites. Table S2. Prevalence of S. mansoni infection according to school attendance in the respective study sites. (DOCX 15 kb) [file 13071_2016_1337_MOESM1_ESM.docx]

SUPPLEMENTARY MATERIAL

**Supplementary table 1.** Prevalence of *S. haematobium* infection according to school attendance in the respective study sites.

|  | | Prevalence of *S. haematobium* infection | |
| --- | --- | --- | --- |
|  |  | School attendance | |
|  |  | Regular | Not regular |
| Study site | Ambatomanambahatse | 0.43 | 0.40 |
|  | Ambodirafia | 1.00 |  |
|  | Ambohibengy | 0.64 | 0.75 |
|  | Ambohimahasoa | 0.00 | 0.00 |
|  | Ambohimahavelona | 0.45 | 0.67 |
|  | Ambohitsara I | 0.88 |  |
|  | Ampamaho | 0.00 | 0.00 |
|  | Ampanihy | 0.22 | 0.11 |
|  | Andohasatra | 0.29 | 0.17 |
|  | Andratamarina | 0.00 |  |
|  | Ankiakabe | 0.00 | 0.00 |
|  | Anoalakely | 0.00 | 0.00 |
|  | Antambiazina | 0.01 | 0.00 |
|  | Antanilojy | 0.02 | 0.08 |
|  | Antapoake | 0.06 | 0.00 |
|  | Antsiradrano | 0.75 | 0.80 |
|  | Bejofo | 0.44 | 0.13 |
|  | Berevo | 0.93 | 0.63 |
|  | Bezavona | 0.00 | 0.00 |
|  | Ekelelahy | 0.08 | 0.07 |
|  | Etanjo | 0.35 | 0.21 |
|  | Makaikely | 0.89 | 1.00 |
|  | Maroanaka | 0.21 | 0.23 |
|  | Miandrarivo II | 0.01 | 0.00 |
|  | Mitia-Est | 0.03 | 0.00 |
|  | Morafeno | 0.28 | 0.19 |
|  | Ranobe | 0.47 | 0.00 |
|  | Soaserana | 0.46 | 0.75 |
|  | Tsaratanana II | 0.72 | 0.75 |

**Supplementary table 1.** Prevalence of *S. mansoni* infection according to school attendance in the respective study sites.

|  | | Prevalence of *S. mansoni* infection | |
| --- | --- | --- | --- |
|  |  | School attendance | |
|  |  | Regular | Not regular |
| Study site | Ambatomanambahatse | 0.00 | 0.00 |
|  | Ambodirafia | 0.00 |  |
|  | Ambohibengy | 0.00 | 0.00 |
|  | Ambohimahasoa | 0.00 | 0.00 |
|  | Ambohimahavelona | 0.03 | 0.00 |
|  | Ambohitsara I | 0.07 |  |
|  | Ampamaho | 0.05 | 0.00 |
|  | Ampanihy | 0.00 | 0.00 |
|  | Andohasatra | 0.00 | 0.00 |
|  | Andratamarina | 0.09 |  |
|  | Ankiakabe | 0.24 | 0.33 |
|  | Anoalakely | 0.07 | 0.20 |
|  | Antambiazina | 0.00 | 0.00 |
|  | Antanilojy | 0.13 | 0.00 |
|  | Antapoake | 0.00 | 0.00 |
|  | Antsiradrano | 0.00 | 0.07 |
|  | Bejofo | 0.02 | 0.00 |
|  | Berevo | 0.00 | 0.06 |
|  | Bezavona | 0.16 | 0.56 |
|  | Ekelelahy | 0.00 | 0.00 |
|  | Etanjo | 0.00 | 0.00 |
|  | Makaikely | 0.02 | 0.00 |
|  | Maroanaka | 0.00 | 0.00 |
|  | Miandrarivo II | 0.05 | 0.00 |
|  | Mitia-Est | 0.87 | 0.75 |
|  | Morafeno | 0.00 | 0.00 |
|  | Ranobe | 0.00 | 0.00 |
|  | Soaserana | 0.00 | 0.00 |
|  | Tsaratanana II | 0.00 | 0.00 |
